# Alleviating Soil Acidification Could Increase Disease Suppression of Bacterial Wilt by Recruiting Potentially Beneficial Rhizobacteria

**DOI:** 10.1128/spectrum.02333-21

**Published:** 2022-03-07

**Authors:** Shuting Zhang, Xiaojiao Liu, Lihua Zhou, Liyuan Deng, Wenzhuo Zhao, Ying Liu, Wei Ding

**Affiliations:** a College of Plant Protection, Southwest University, Chongqing, China; University of Massachusetts Amherst

**Keywords:** soil acidification, bacterial wilt, bacterial communities, beneficial bacteria

## Abstract

Bacterial wilt is accompanied by microbial communities shift and soil acidification. However, the relationship between the changes of bacterial communities and bacterial wilt under the influence of different acidification levels has not been fully elucidated. Here, we analyzed the abundance of Ralstonia solanacearum, rhizosphere bacterial communities and carbon metabolism at differently acidic levels (pH 6.45, pH 5.60, pH 5.35, pH 4.90 and pH 4.45) and soil amendment treatment (CaO). The results indicated that both the abundance of R. solanacearum and the incidence of bacterial wilt showed a significant trend of first increasing and then decreasing with the increase of soil pH. The Firmicutes phylum and potentially beneficial genera *Bacillus*, *Paenibacillus*, *Flavobacterium* and Pseudomonas were significantly enriched at pH 6.45. The metabolic ability in response to the l-arginine and 4-hydroxybenzoic acid was significantly increased at pH 6.45. After using CaO to increase the pH of diseased soil from 5.45 to 6.05, the abundance of R. solanacearum and the incidence of bacterial wilt were significantly reduced, the Firmicutes and potentially beneficial genera *Bacillus* and Pseudomonas were significantly enriched. Overall, alleviating soil acidification to a slightly acidic level (pH 6.0–6.5) could suppress bacterial wilt by suppressing the growth of R. solanacearum and enriching the rhizosphere potentially beneficial bacteria, and further emphasized the importance of increasing soil pH in biological control of bacterial wilt.

**IMPORTANCE** The rhizosphere microbiota and soil acidification have been shown to have impacts on bacterial wilt. However, the influence of different acidification levels on the rhizosphere communities and bacterial wilt has not been fully studied. In this study, the potentially beneficial bacteria (*Bacillus* and Pseudomonas) were significantly enriched in the slightly acidic soil (pH 6.45), leading to the increase of the metabolism of 4-hydroxybenzoic acid and the decrease of pathogenic R. solanacearum, thereby alleviating the occurrence of bacterial wilt. The changes of potentially beneficial bacteria and pathogenic R. solanacearum in strongly acidic soil (pH 5.35) with the highest incidence of bacterial wilt were just the opposite. These findings help clarify the mechanisms by which soil bacteria exert influence on bacterial wilt outbreak under different soil acidification levels.

## INTRODUCTION

Excess soil acidification is a major problem in worldwide soil deterioration and is becoming increasingly serious in intensive agriculture (1). To better understand the cation‐anion pools in soil, different ranges of soil pH have been employed to determine the variation of soil acidity. Generally, most crops favor soils with pH between 5.5 and 6.5, which belongs to slightly acid (pH 6.0–6.5) and moderately acid (pH 5.5–6.0) (2, 3). However, strongly acidic soil (pH 4.5–5.5) represents 30%–40% of the world’s arable soils, and adversely affects the production of many crops (3, 4). At pH 4.5 or below (extremely acid), the Al^3+^ predominates in the soil solution and has the greatest impact on plant growth (5). In addition, many researchers revealed that soil acidification is closely related to the occurrence of soilborne disease (6–9).

Bacterial wilt, caused by Ralstonia solanacearum, is a typical soilborne disease that can infect *Solanac*eae crops (10), such as tobacco (11), tomato (12), and eggplant (13). Previous studies showed that the occurrence of bacterial wilt was related to soil pH. Strongly acidic condition (pH 4.5–5.5) was conductive to R. solanacearum growth in B medium, which aggravated the occurrence of bacterial wilt in the pot experiment (6). Within the range of pH 4.5–6.5, pH had a significantly negative correlation with bacterial wilt infection rate (14). Meanwhile, the occurrence of bacterial wilt can be effectively controlled by anthropogenically increasing soil pH (15–17). Earlier studies indicated that soil amendments calcium oxide (18), rock dust (19) and calcium carbonate (20) were effective for controlling bacterial wilt by increasing the soil pH. However, how soil pH affects the occurrence of bacterial wilt is still unclear.

On the other hand, studies have shown that the occurrence of bacterial wilt is also closely related to the bacterial community composition of rhizosphere soil (11, 14, 21). Gram-positive bacteria Firmicutes and Actinobacteria, have been identified as bacterial wilt disease-suppressing rhizobacteria (22). Meanwhile, diverse beneficial rhizobacterial genera have been identified as disease-suppressing microbes, including the genera of *Bacillus* (23, 24), Pseudomonas (25), *Streptomy*ces (23), *Paenibacillus* (26), *Flavobacterium* (27), and *Arthrobac*ter (28). Moreover, in bacterial wilt-suppression soil, the enrichment of beneficial microbes in soil was closely related to the metabolism of l-arginine and 4-hydroxybenzoic acid (an auto-toxic substance secreted by plant root) (29).

Changes in soil pH can strongly affect the activity and community structure of soil microorganisms (30). And bacterial communities were more strongly influenced by pH than fungal communities (31). Therefore, the occurrence of bacterial wilt is closely related to soil pH and bacterial community composition. However, the bacterial community composition under different soil acidity is still unclear together with the relationship in the suppression of bacterial wilt disease. In this study, we changed the soil acidity of nondiseased soil (experiment I, Fig. S1), then soil amendment CaO (experiment II) was used to improve the pH of acidic bacterial wilt diseased soil, investigated i) the changes in bacterial community composition at different acidity levels, and ii) the relationship between the change in bacterial community composition and the occurrence of bacterial wilt.

## RESULTS

### Soil chemical properties and incidence of bacterial wilt in experiment I.

The changes in soil chemical properties in response to different soil pH are shown in Table 1. With the decrease of soil pH, the contents of available nitrogen and exchangeable aluminum were significantly increased (*P* < 0.05), while the exchangeable calcium was significantly decreased (*P* < 0.05). Moreover, the contents of available phosphorus and exchangeable magnesium decreased first and then increased.

**TABLE 1:** The basic chemical properties at different pH levels^*a*^

Treatment	SOM (g/kg)	AN (mg/kg)	AP (mg/kg)	AK (mg/kg)	AS (mg/kg)	ExMg (mg/kg)	ExCa (mg/kg)	ExAl (mg/kg)
pH4.45	31.40 ± 0.05a	161.98 ± 1.03d	38.67 ± 0.23c	254.16 ± 2.89b	86.82 ± 1.83c	64.50 ± 1.50c	961.50 ± 15.08a	302.89 ± 1.62e
pH4.90	36.55 ± 0.08a	142.04 ± 1.44c	31.23 ± 0.27b	255.22 ± 0.29b	85.19 ± 3.53c	65.00 ± 2.02c	952.50 ± 8.80a	238.15 ± 1.85d
pH5.35	30.04 ± 0.06a	139.55 ± 0.01c	29.46 ± 0.38b	214.40 ± 1.39a	63.54 ± 1.73a	46.75 ± 0.35a	990.00 ± 3.37b	76.97 ± 0.79b
pH5.60	33.99 ± 0.08a	130.83 ± 2.16a	24.78 ± 0.16a	285.38 ± 2.99d	73.55 ± 5.45b	50.67 ± 2.45ab	1036.67 ± 12.25b	73.35 ± 1.04b
pH6.45	31.48 ± 4.47a	132.08 ± 1.18ab	33.07 ± 2.68bc	266.14 ± 0.19c	65.94 ± 1.26a	58.25 ± 0.19b	1102.50 ± 2.80c	40.29 ± 0.64a

a The results are the mean of three measurement replicates ± standard error. Small letters indicate a significant difference among different samples (one-way ANOVA, *P* < 0.05). SOM, soil organic matter; AN, available nitrogen; AP, available phosphorus; AK, available potassium; AS, available sulfur; ExMg, exchangeable magnesium; ExCa, exchangeable calcium; ExAl, exchangeable aluminum.

**FIG 1:**
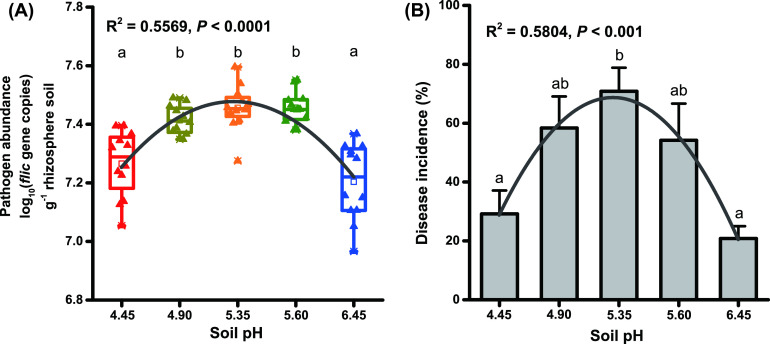
The occurrence of bacterial wilt at different acidification gradients. (A) The abundance of pathogen R. solanacearum in different samples. (B) The disease incidence of different treatments. Different letters indicate significant (*P* < 0.05) differences according to one-way ANOVA.

The abundance of R. solanacearum in the rhizosphere soil of pH 4.90 – pH 5.60 was significantly higher than that of pH 4.45 and pH 6.45. And in the acidic range, the abundance of R. solanacearum in the rhizosphere soil showed a trend of increased firstly and then decreased with the increase of soil pH (R^2^ = 0.5569, *P* < 0.0001, Fig. 1A). Correspondingly, the incidence of tobacco bacterial wilt also increased first and then decreased with the increase of soil pH (R^2^ = 0.5804, *P* < 0.001). Compared with pH 4.45 and pH 6.45, pH 5.35 significantly increased the disease incidence of bacterial wilt by 41.66% and 50.00%, respectively (Fig. 1B, Fig. S2).

### Composition of bacterial community at different acidity levels in experiment I.

**FIG 2:**
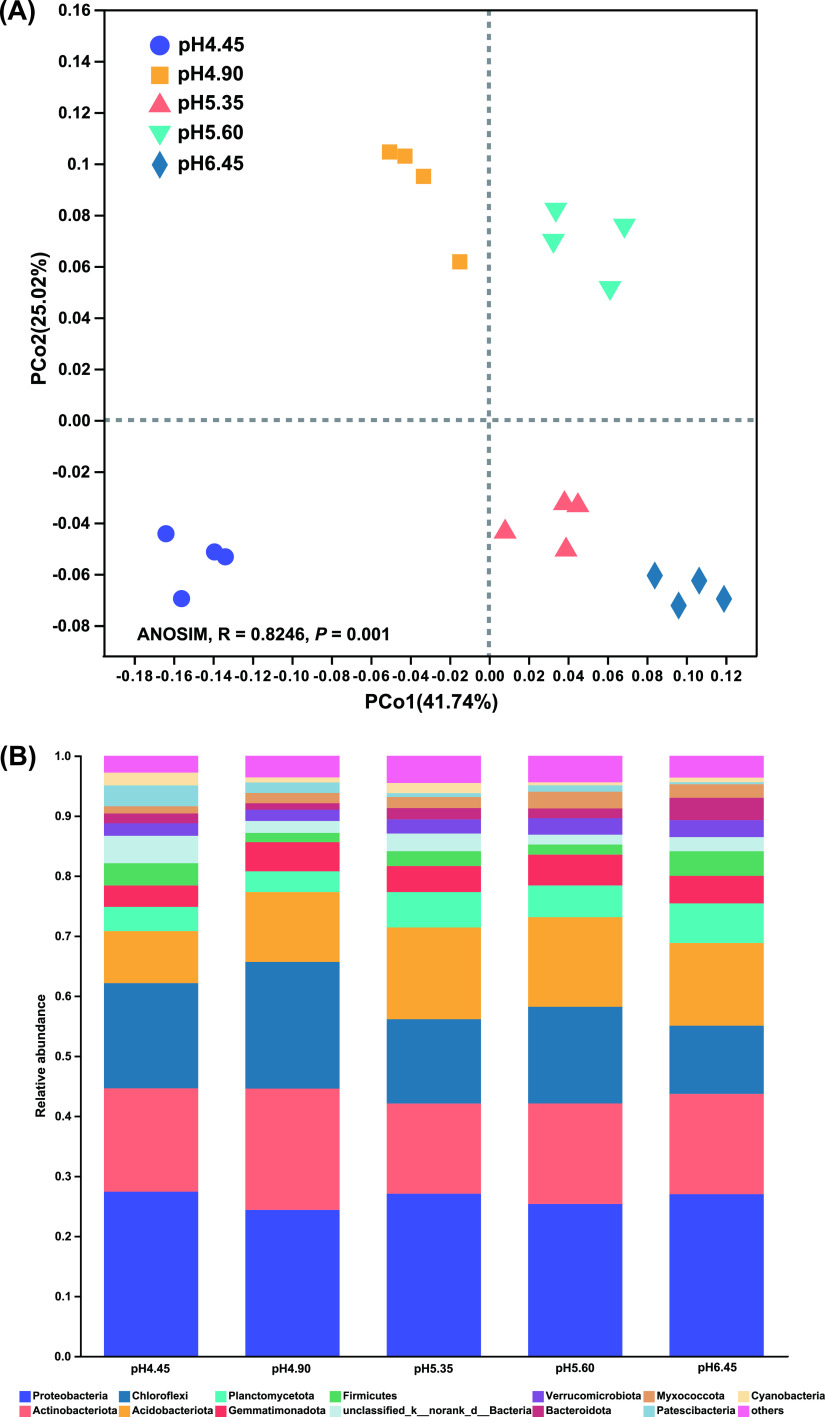
Comparison of soil microbial community structure among different samples. (A) Principal coordinates analysis (PCoA) by weighted UniFrac of bacterial composition from different soil acidification levels. (B) Relative abundance of bacterial phyla with an abundance greater than 1%.

The rarefaction curve showed that the sequencing efforts of bacteria were sufficient for this study as the number of ASVs was saturated (Fig. S3). The bacterial community structure was illustrated using a PCoA plot based on the weighted UniFrac index. The bacterial communities were significantly different among the pH 4.45, pH 4.90, pH 5.35, pH 5.60 and pH 6.45 samples (*R* = 0.8246, *P* = 0.001, ANOSIM) (Fig. 2A).

All bacterial communities were dominated by phyla Proteobacteria, Actinobacteriota, Acidobacteriota, and Chloroflexi with 24.37–27.41%, 15.03–20.20%, 11.35–21.09% and 8.68–15.28% average relative abundance, respectively. The relative abundance of Firmicutes at pH 6.45 was the highest (4.10%), followed by pH 4.45 (3.71%). There was no significant difference in the relative abundance of Firmicutes between pH 6.45 and pH 4.45, however, the relative abundance was significantly higher than pH 4.90 (1.55%), pH 5.35 (2.48%) and pH 5.60 (1.69%) (Fig. 2B).

**FIG 3:**
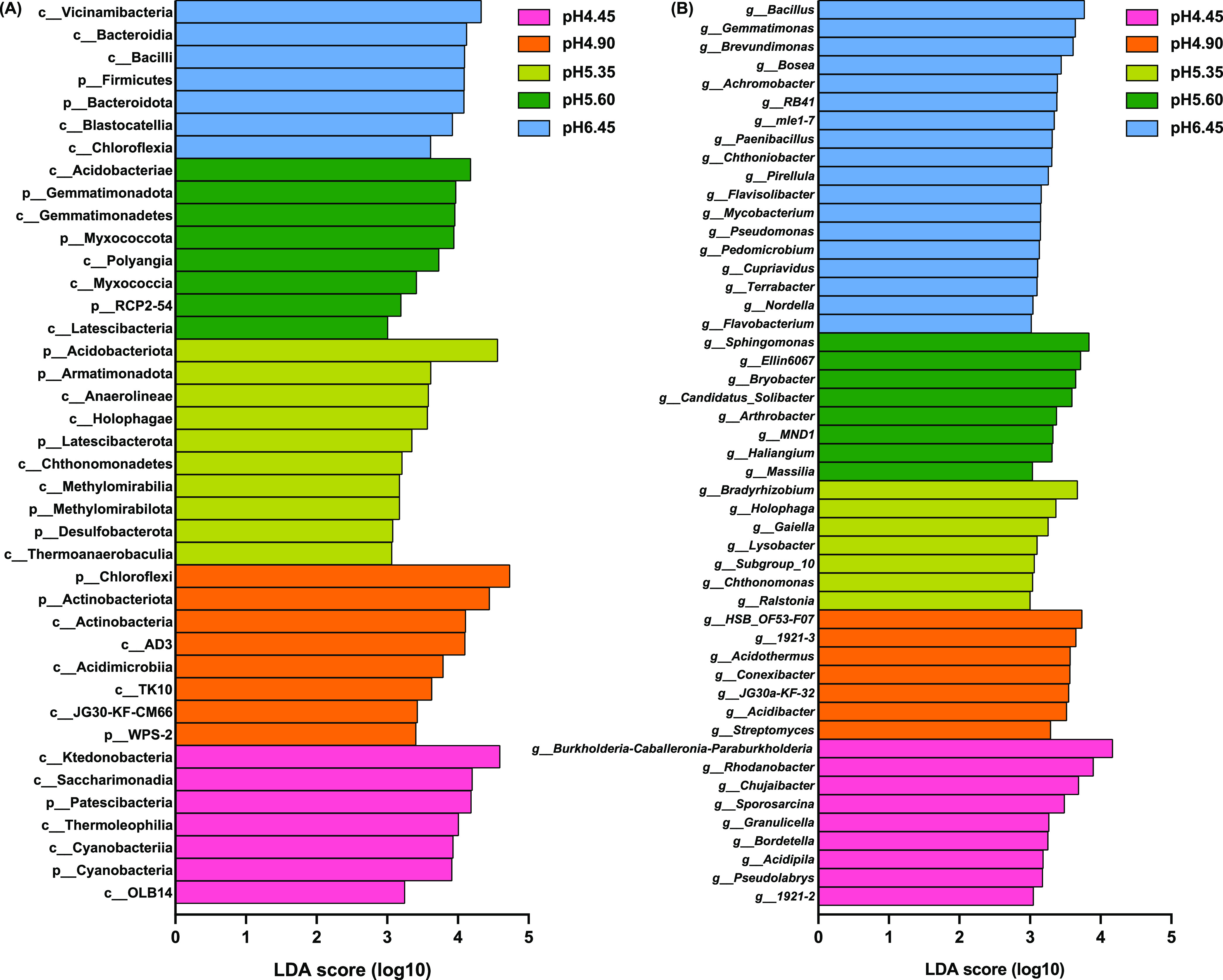
Histogram of the LDA scores computed for differentially abundant bacterial phyla and classes (A) and genera (B, deleted the norank and unclassified taxa) under different acidification levels.

The linear discriminant analysis (LDA) effect size (LEfSe) method was used to detect bacterial taxa causing significant differences at the different pH levels. At the phylum level, Patescibacteria (LDA = 4.19) and Cyanobacteria (LDA = 3.92) were enriched at pH 4.45. Chloroflexi (LDA = 4.73), Actinobacteriota (LDA = 4.45) and WPS-2 (LDA = 3.41) were enriched at pH 4.90. Acidobacteriota (LDA = 4.56), Armatimonadota (LDA = 3.62), Latescibacterota (LDA = 3.35), Methylomirabilota (LDA = 3.17) and Desulfobacterota (LDA = 3.08) were enriched at pH 5.35. Gemmatimonadota (LDA = 3.97), Myxococcota (LDA = 3.95) and RCP2-54 (LDA = 3.20) were enriched at pH 5.60. Firmicutes (LDA = 4.09) and Bacteroidota (LDA = 4.09) were enriched at pH 6.45 (Fig. 3A).

**FIG 4:**
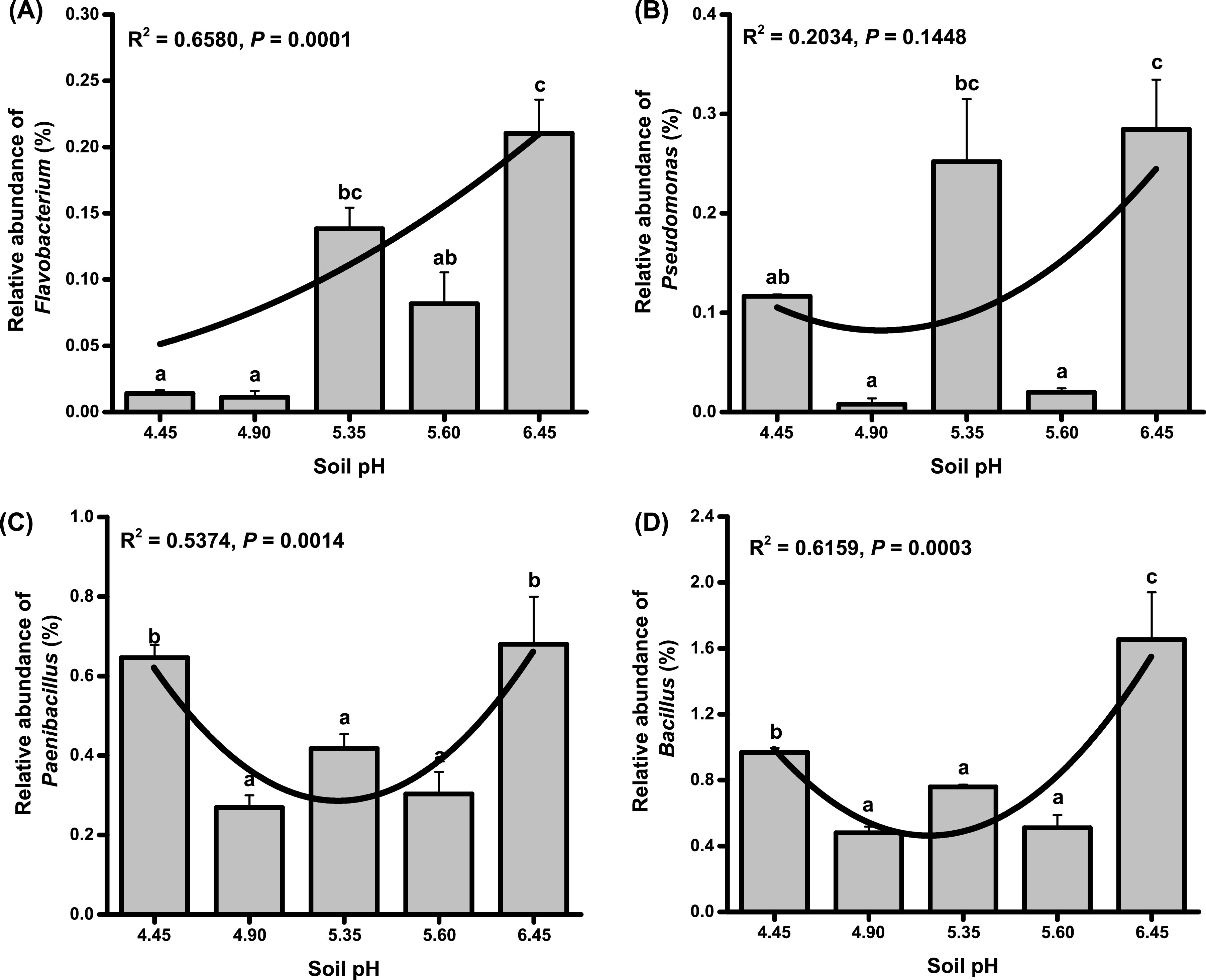
The relative abundance of *Flavobacterium* (A), Pseudomonas (B), *Paenibacillus* (C), and *Bacillus* (D) at different acidification levels. Different letters indicate significant (*P* < 0.05) differences according to one-way ANOVA.

At the genus level (removing norank and unclassified taxa), *Ralstonia* (LDA = 3.00) was enriched at pH 5.35. Whereas *Bacillus* (LDA = 3.77), *Paenibacillus* (LDA = 3.32), Pseudomonas (LDA = 3.15) and *Flavobacterium* (LDA = 3.02) were significantly enriched at pH 6.45 (Fig. 3B). In the range of acid treatment, with the increase of soil pH, the relative abundance of *Flavobacterium* increased significantly (R^2^ = 0.6580, *P* = 0.0001), and the relative abundance of *Paenibacillus* (R^2^ = 0.5374, *P* = 0.0014) and *Bacillus* (R^2^ = 0.6159, *P* = 0.0003) showed a significant trend of first decreasing and then increasing (Fig. 4). Moreover, spearman correlation analysis showed that there was an extremely significant negative correlation between the relative abundance of *Paenibacillus* (*r* = −0.708, *P* = 0.0004) and *Bacillus* (*r* = −0.749, *P* = 0.0005) with the abundance of R. solanacearum.

### Relationships between shifts in the bacterial community composition and environmental variables in experiment I.

The effects of environmental variables on the bacterial communities were assessed by redundancy analysis (RDA) (Fig. S4). AN (R^2^ = 0.9295, *P* = 0.001), ExAl (R^2^ = 0.8811, *P* = 0.001), soil pH (R^2^ = 0.7899, *P* = 0.001), AS (R^2^ = 0.6885, *P* = 0.001), AP (R^2^ = 0.6708, *P* = 0.002), ExCa (R^2^ = 0.6012, *P* = 0.001) and ExMg (R^2^ = 0.3451, *P* = 0.026) were significantly correlated with bacterial community structures at the different soil pH levels (Fig. S4, Table S1).

### Carbon metabolism of the microbial community at different acidity levels in experiment I.

**FIG 5:**
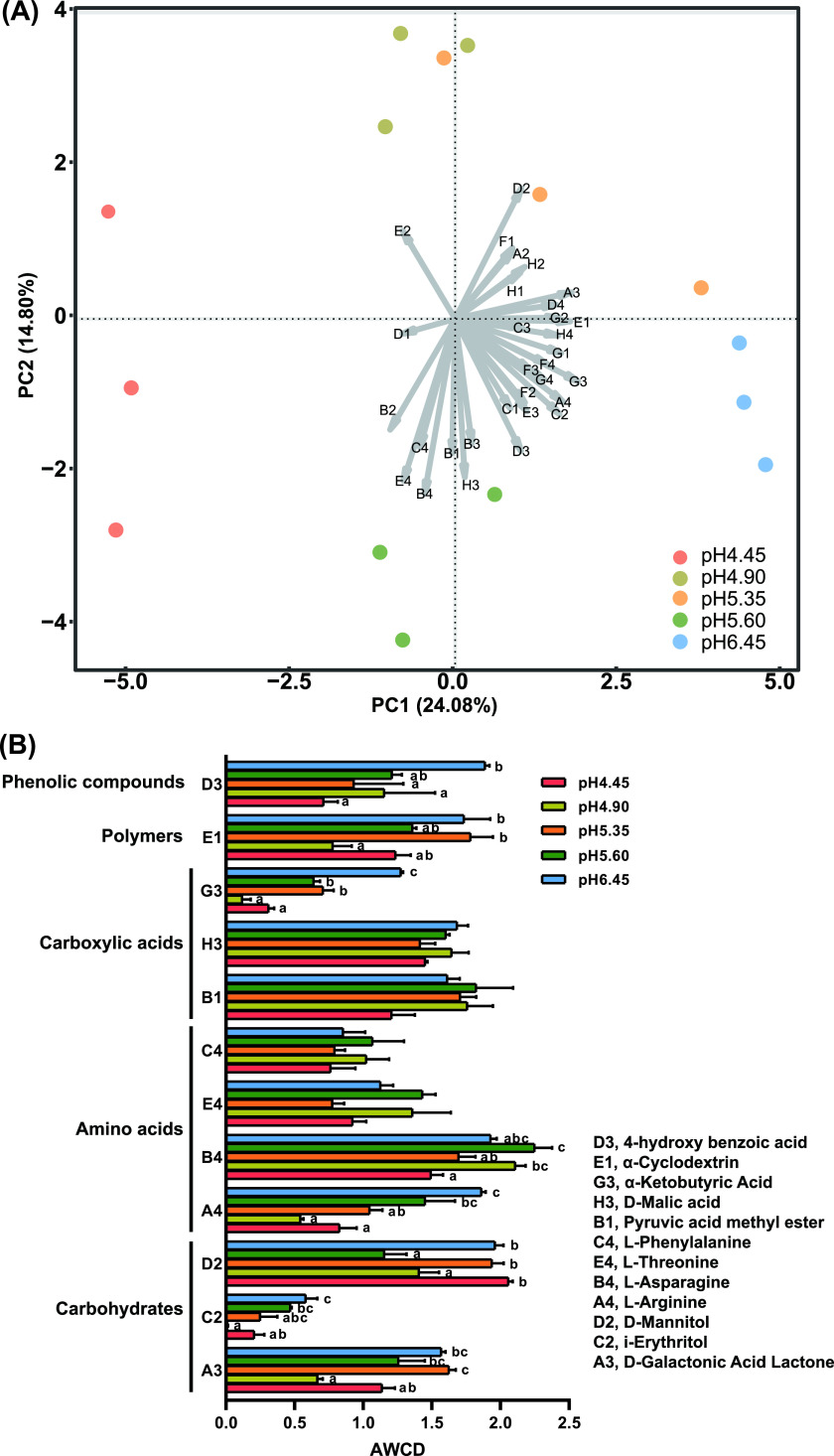
Microbial carbon source metabolism capacity at different soil pH. A, PCA ordination biplot of the different pH levels according to their carbon source utilization profile. All carbon sources are indicated by arrows. Longer arrows indicate a greater change in carbon source utilization value. B, The average well color development (AWCD) among different treatments (carbon sources which had the arrow length above the average). Different letters indicate significant (*P* < 0.05) differences according to one-way ANOVA. A2, β-Methyl-d-Glucoside; B2, d-Xylose; B3, d-Galacturonic Acid; C1, Tween 40; C3, 2-Hydroxybenzoic Acid; D1, Tween 80; D4, L-Serine; E2, N-Acetyl-d-Glucosamine; E3, γ-Hydroxybutyric Acid; F1, Glycogen; F2, d-Glucosaminic Acid; F3, Itaconic Acid; F4, Glycyl-L-Glutamic Acid; G1, d-Cellobiose; G2, α-d-Glucose-1-Phosphate; G4, Phenylethylamine; H1, α-d-Lactose; H2, D, L-α-Glycerol Phosphate; H4, Putrescine.

The average well color development (AWCD) values in pH 6.45 were the highest, and those at pH 4.45 were the lowest. As the soil pH increased, the carbon source metabolism capacity of soil microorganisms increased (Fig. S5). In the PCA of the Biolog data at 72 h, the microbial carbon source metabolism was significantly different between pH 4.45 and pH 6.45 (Fig. 5A). Carbon sources that had the arrow length above the average in Fig. 5A were selected to further present their well color development among different treatments in Fig. 5B. With the increase of soil pH, the metabolic capacity of 4-hydroxybenzoic acid, α-ketobutyric acid, l-arginine and i-erythritol were significantly enhanced.

### Effect of CaO on the occurrence of bacterial wilt and the rhizosphere bacterial communities in experiment II.

**FIG 6:**
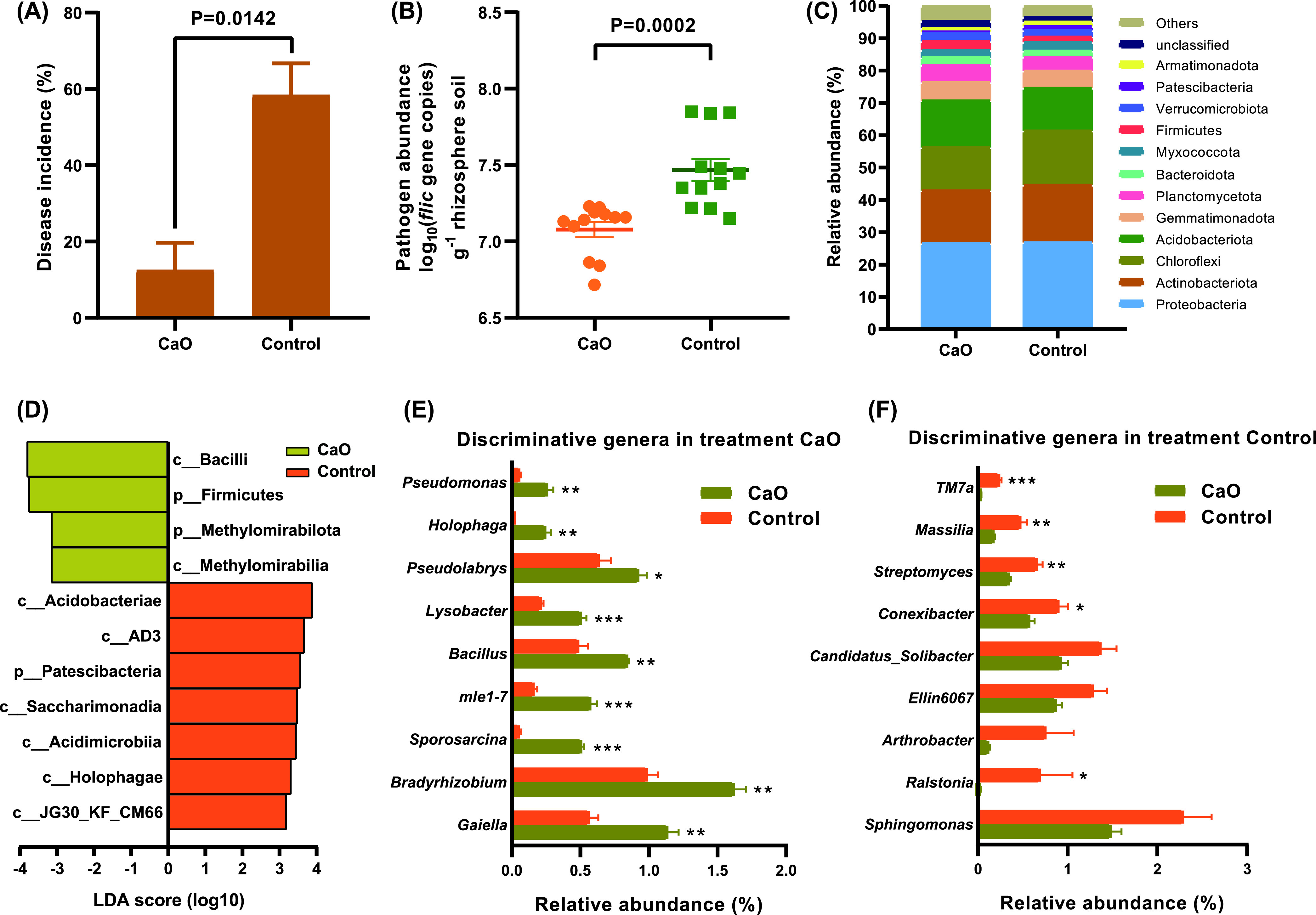
The occurrence of bacterial wilt and comparison of soil community structure between CaO and control in diseased soil. (A) The effect of CaO treatment on the incidence of bacterial wilt. (B) The influence of CaO treatment on the abundance of pathogen R. solanacearum. (C) The relative abundance of bacterial taxa at phylum level. (D) Histogram of the LDA scores computed for differentially abundant bacterial phyla and classes in CaO and control samples. (E–F) The relative abundances of discriminative genera (LDA > 3.0) in CaO and control samples, respectively. The *P* value and asterisks indicate significantly (* 0.01 < *P* ≤ 0.05, ** 0.001 < *P* ≤ 0.01, ***, *P* ≤ 0.001) difference between CaO and control, as determined by independent-sample *t*-test.

After adding CaO to the acidic soil, the soil pH increased from 5.45 to 6.05. Compared with the control, the disease incidence of bacterial wilt (*P* = 0.0142, independent-sample *t*-test) and the abundance of R. solanacearum (*P* = 0.0002, independent-sample *t*-test) in CaO treatment were significantly decreased by 45.83% and 1.05-fold, respectively (Fig. 6A and B).

Principal coordinate analysis, based on the Bray–Curtis dissimilarity index, revealed clear differences between CaO and control samples (Fig. S6). Relative abundance analysis indicated that Proteobacteria (26.58–26.81% average relative abundance), Actinobacteriota (16.31–17.80% average relative abundance), Chloroflexi (13.35–16.80% average relative abundance), and Acidobacteriota (13.19–14.48% average relative abundance) were the main bacterial communities at the phylum level. The relative abundance of Firmicutes in CaO treatment was significantly increased by 1.66-fold compared with the control (Fig. 6C). Moreover, Firmicutes (LDA = 3.75), Methylomirabilota (LDA= 3.15), Bacilli (LDA = 3.80) and Methylomirabilia (LDA = 3.14) were significantly enriched in CaO (Fig. 6D). At the genus level (removing norank and unclassified taxa), *Bacillus* (LDA = 3.24) and Pseudomonas (LDA = 3.00) were significantly enriched in CaO, and the relative abundances were significantly increased by 1.75-fold and 4.56-fold compared with control, respectively (Fig. 6E). Whereas *Ralstonia* (LDA = 3.52) was significantly enriched in control, and the relative abundance was increased by 34.50-fold compared with CaO treatment (Fig. 6F).

CaO treatment increased the microbial carbon source metabolism capacity of rhizosphere soil microorganisms (Fig. S7A). The AWCD of L-arginine and 4 – hydroxybenzoic acid in CaO was higher than that of control, however, there was no significant difference between CaO and control (Fig. S7B-C).

## DISCUSSION

### Soil slightly acidic environment can alleviate the occurrence of bacterial wilt.

Strongly acidic soil (pH 4.5–5.5) is beneficial to the growth of R. solanacearum and aggravates the occurrence of bacterial wilt (6, 32). Some of the control measures of bacterial wilt, such as the addition of biochar (increasing the soil pH from 4.90 to 6.20), rock dust (increasing the soil pH from 5.13 to 6.81) and lime (increasing the soil pH over 6.00), one of the effective factors is improve the soil from strong acidity to slight acidity (pH 6.0–7.0) (2, 6, 15, 19). For some *solanace*ous crops, the optimum growth pH of tobacco is 6.0 (33), and tomato is pH 6.0–6.8 (34). In this study, the incidence of bacterial wilt with pH 6.45 was the lowest (Fig. 1B). CaO treatment increased the soil pH from 5.45 to 6.05, and compared with control (pH 5.55), the incidence of bacterial wilt was significantly reduced (Fig. 6A). The results showed that when using soil amendments to increase soil pH to control tobacco bacterial wilt, the soil pH is preferably at a slightly acidic level (between 6.0 and 6.5).

### Beneficial bacteria increased the soil suppression of bacterial wilt in slightly acidic soil.

Because a steady-state balance of microbial community composition is essential for healthy host-microbe relationships, the enrichment and disruption of the microbial community is an important mechanism for the occurrence of plant diseases (35, 36). Firmicutes and Actinobacteriota abundance in the tomato rhizosphere conferred suppression of bacterial wilt (22). Firmicutes had a positive correlation with plant immunity (37). In this study, compared with pH 6.45, the relative abundance of Firmicutes was significantly decreased at pH 5.35 (Fig. 2B). Moreover, Firmicutes was significantly enriched at pH 6.45 (Fig. 3A), and after CaO (pH 6.05) was used to increase the pH, Firmicutes was also significantly enriched (Fig. 6D).

*Bacillus* (38–40) and Pseudomonas (41, 42) have been extensively studied for the growth promotion and suppression of bacterial wilt caused by R. solanacearum. Moreover, *Bacillus* and Pseudomonas were negatively related to the abundance of R. solanacearum (43). In this study, *Bacillus* had an extremely significant negative correlation with the R. solanacearum, however, there was no significant correlation between the Pseudomonas and R. solanacearum, which may be related to the effect of soil pH. The strains of *Paenibacillus* have been widely used for the control of bacterial wilt (44, 45). And the *Flavobacterium* in the rhizosphere of bacterial wilt resistant plants was much higher than that of susceptible plants (27). In this study, the abundance of R. solanacearum was significantly decreased at pH 6.45 (Fig. 1A), the potentially beneficial bacteria, *Bacillus*, *Paenibacillus*, Pseudomonas and *Flavobacterium* were significantly enriched at pH 6.45 (Fig. 3B). Moreover, after increasing soil pH by CaO (pH 6.05), the abundance of R. solanacearum also decreased significantly (Fig. 6B), and the relative abundance of *Bacillus* and Pseudomonas increased significantly (Fig. 6E). These results suggested that slightly acidic soil could increase the abundance of potentially beneficial bacteria and decrease the pathogen abundance in the rhizosphere, which led to the dominance of beneficial bacteria in the rhizosphere soil, and thus increased the soil suppression of bacterial wilt.

### Microorganisms in slightly acidic soil increased their ability to metabolize specific carbon sources.

Soil environments are usually oligotrophic, where microbes compete fiercely for limited nutrients, such as carbon sources (46). Biolog can be used as an indicator of the microbial potential for carbon sources usage and potential changes therein as the results of the changes in environmental conditions (47). l-arginine can not only promote the growth of Bacillus amyloliquefaciens, but also promote the production of antibiotics bacillaene and macrolactin by Bacillus amyloliquefaciens, thereby inhibiting the growth of bacterial wilt (48). The excessive accumulation of 4-hydroxybenzoic acid in the soil inhibits the growth of crops, leading to crop yield reduction, continuous cropping obstacles and destruction of the natural ecological environment (49, 50). 4-hydroxybenzoic acid is one of the major autotoxins secreted by plant roots (51), and (52) indicated that 4-hydroxybenzoic acid was a strong chemoattractant for R. solanacearum. In addition, studies have shown that *Thermophilic Bacillus* sp. (53) and Pseudomonas sp. (54) can effectively degrade 4-hydroxybenzoic acid in the soil. l-arginine and 4-hydroxybenzoic could not be metabolized by R. solanacearum, whereas they were significantly metabolized in disease-suppressive of bacterial wilt soils, and they may act as indicators for deciphering the bacterial wilt suppression pattern (29). All in all, l-arginine and 4-hydroxybenzoic may promote the growth of the beneficial microbes, instead of the pathogen R. solanacearum. In this study, the microorganisms at pH 6.45 and CaO treatment increased their metabolic ability to the l-arginine and 4-hydroxybenzoic acid (Fig. 5B, Fig. S7B-C). These may be related to the enrichment of the potentially beneficial bacteria, such as *Bacillus* and Pseudomonas.

### Soil pH affected rhizosphere bacterial community composition by changing soil chemical properties.

The forces that shape the rhizosphere microbial community cannot be fully understood without discussing the influence of the soil environment (55). Soil variables had an additional, indirect effect on the rhizosphere bacterial communities due to their influence on the composition of the bulk soil bacterial communities (56). In addition, specific soil physicochemical conditions of bulk soil, especially soil pH, may directly select for particular bacterial species in the rhizosphere (57). Soil pH can also affect the rhizosphere bacterial community structure indirectly by influencing nutrient availability (58, 59). And the availability of elements in the soil is closely related to soil pH (2). In this study, soil pH, available nitrogen, available phosphorus, available sulfur, exchangeable calcium, exchangeable magnesium and exchangeable aluminum were significantly correlated with bacterial communities. Moreover, the effects of available nitrogen and exchangeable aluminum ions on soil bacterial communities are stronger than soil pH (Fig. S4). Soil nitrogen availability and soil pH identified as the two most influential soil properties to influence the soil microbial community composition under nitrogen deposition (60). Yang et al. (61) indicated that nitrogen-induced changes in soil pH are an important mechanism driving the ecosystem functions. When the soils with a pH of 5.5 or lower, aluminum ions are dissolved from clay minerals, and there is a significant negative correlation between the aluminum ions and the soil pH (5, 62). These results suggested that soil pH may affect the microbial community composition by changing the availability of soil elements.

### An extremely acidic soil environment also reduced the occurrence of bacterial wilt.

Aluminum ions have a toxic effect on the growth of plants (3). The organic acids released from plant roots, such as citric acid, oxalic acid, and malic acid, can chelate Al^3+^, thereby alleviating aluminum toxicity (63). Aluminum stress changed the rhizosphere bacterial communities (64). When plants are in a stressful environment, root exudates can attract beneficial microbes from the environment, which is called a “cry for help” strategy (65). Aluminum stress can stimulate the increase of *Bacillus* and Pseudomonas to alleviate ginger aluminum toxicity and bacterial wilt in extremely acidic soil (pH less than 4.5) (7). In this study, in a high-aluminum soil environment with pH 4.45, the growth of tobacco was not significantly affected (Fig. S2). In the acidic range, the abundance of R. solanacearum and the incidence of bacterial wilt showed a significant trend of first increasing and then decreasing with the increase of soil pH (Fig. 1). However, the relative abundances of *Paenibacillus* and *Bacillus* showed opposite trends (Fig. 4C and D). This may be the result of the release of root exudates from tobacco to alleviate aluminum toxicity while increasing the relative abundance of potentially beneficial bacteria in the rhizosphere. We are studying the relationship between aluminum stress and bacterial wilt.

**FIG 7:**
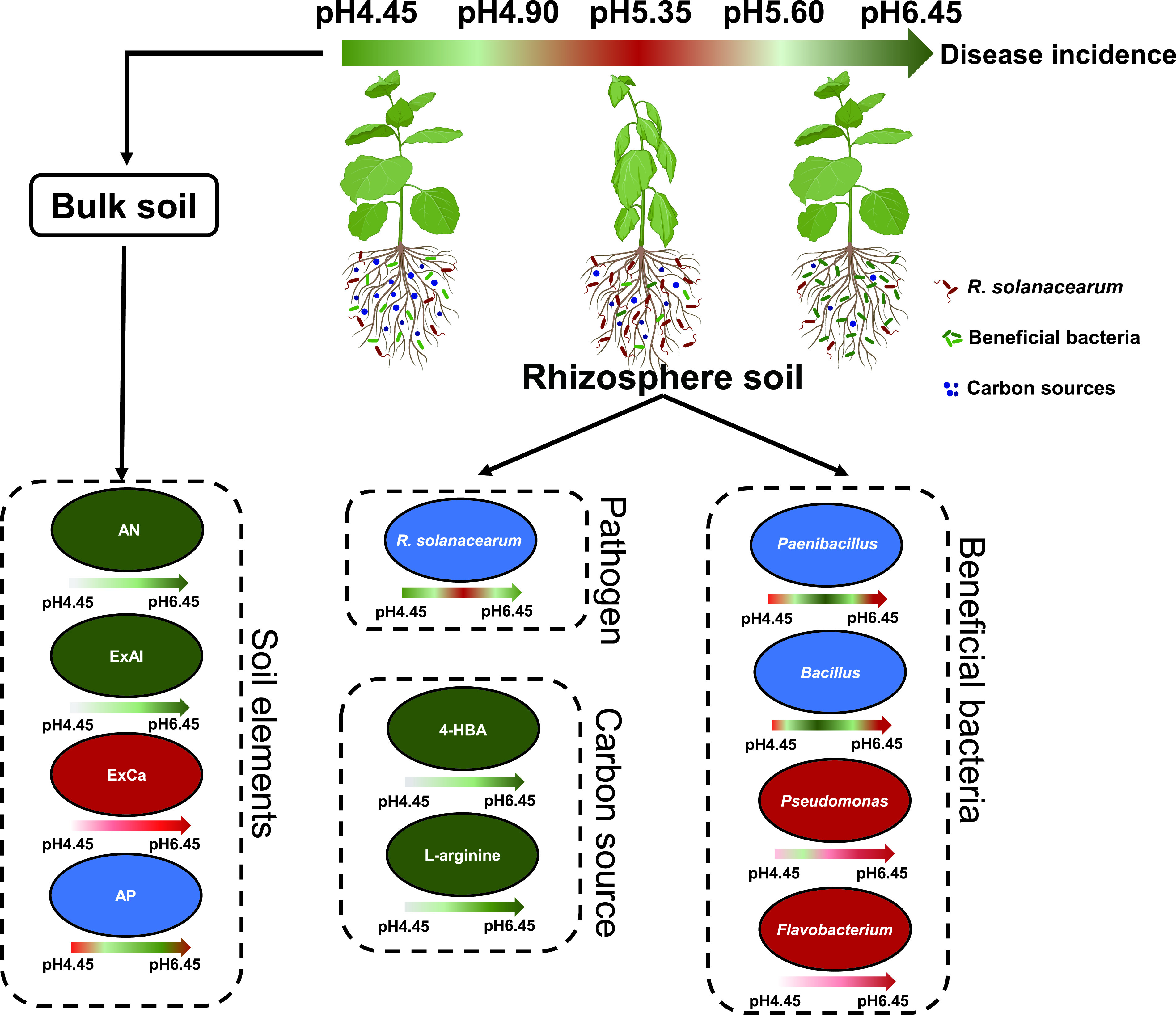
The influence of different acidification levels on various factors. The green color indicates downregulation, the red color indicates upregulation, and the blue color indicates both upregulation and downregulation. The intensity of the color in the arrows is proportional to the extent of the changes. AN, AP, ExCa, and ExAl indicate available soil nitrogen, available phosphorus, soil exchangeable calcium, and exchangeable aluminum, respectively. 4-HBA indicates 4-hydroxybenzoic.

In conclusion, slightly acidic soil (pH 6.45) and extremely acidic soil (pH 4.45) suppressed the growth of pathogenic R. solanacearum, thereby alleviating the occurrence of bacterial wilt. Moreover, changes in soil elements availability associated with soil acidic level significantly affected the soil bacterial community structure, leading to the enrichment of the potentially beneficial bacteria and the increase of the metabolism of 4-hydroxybenzoic acid in the slightly acidic soil (pH 6.45), and further the suppression of bacterial wilt (Fig. 7). These findings also explain that biological control of bacterial wilt by adding *Bacillus* or Pseudomonas, adjusting soil pH to a slightly acidic condition (pH 6.0–6.5) is the prerequisite to achieve a better control effect.

## MATERIALS AND METHODS

The effects of differently acidic pH gradients on tobacco rhizosphere microbial community composition were studied in two pot experiments. In the first experiment, nondiseased soil without bacterial wilt was adjusted to different acid gradients. Based on the results of the first experiment, the effect of soil amendment on the rhizosphere bacterial community by increasing the pH of diseased soil with bacterial wilt was further studied in the second experiment.

### Soil sampling.

According to the survey, soil without bacterial wilt for continuous cropping 5 years was considered nondiseased soil, whereas the occurrence of bacterial wilt every year for 5 continuous years was considered diseased soil. Nondiseased (107°57.913′ E, 29°10.008′ N, 1315 m) and diseased (107°56.618′ E, 29°08.291′ N, 1219 m) soil samples were collected from Pengshui in Chongqing city, China, in May 2020. The nondiseased (sand-clay-silt, 32.57%–3.56%-63.87%) and diseased (sand-clay-silt, 21.75%–3.67%-74.58%) soil were classified as silt loam (32). Samples were obtained from the 10–20 cm of the soil. The soil samples were filtered through a 2 mm mesh to remove large soil particles and plant root tissue and debris. The soils were temporarily stored at 4°C for subsequent experiments.

### Experimental setup.

Experiment I, nondiseased soil was treated with 0.1 mol/L NaOH or H_2_SO_4_ to adjust the soil pH to extreme acidity (pH below 4.5), very strong acidity (pH 4.5–5.0), strong acidity (pH 5.0–5.5), moderate acidity (pH 5.5–6.0) and slight acidity (pH 6.0–6.5). During the cultivation period, the pH was checked every 2 days to keep the pH within the set pH range and to keep the soil water holding capacity at 60%. After 30 days of incubation, the pH of the samples was 4.45, 4.90, 5.35, 5.60 and 6.45, and 500 g of soil was collected for chemical property analysis. Nicotiana benthamiana seedlings were then planted in the soil as the first population (1°, *n* = 32 plants per treatment). The rhizosphere soil of the plants (1°) was taken after 20 days of growth (each treatment had 4 replicates, and each replicate took rhizosphere soil from 8 tobacco seedlings), and the soil samples were stored at −20°C until needed for DNA extraction. Then the second population (2°) of Nicotiana benthamiana was planted in bulk soil with different pH levels (*n* = 24 plants per treatment). After 7 days of growth, 10 mL of Ralstonia solanacearum wild-type strain CQPS-1 (66) with OD_600_ = 0.01 was added to each tobacco seedling, and then the disease incidence of bacterial wilt was investigated after 14 days (Fig. S1).

Experiment II, the initial pH of the diseased soil was 5.45. The diseased soil was equally divided into 2 parts, and then the soil was treated with 1 g/kg of CaO and deionized water (control), respectively. After 30 days of soil treatment, the pH of the soil with CaO and control was 6.05 and 5.55, respectively. Before planting Nicotiana benthamiana seedlings, a small amount of soil was collected for the quantitative detection of R. solanacearum. Then, Nicotiana benthamiana seedlings were planted to investigate the occurrence of bacterial wilt (*n* = 24). On the 15th day after planting tobacco seedlings, the incidence of bacterial wilt was determined, and then the rhizosphere soil of tobacco seedlings was collected and stored at −20°C for DNA extraction.

### Determination of soil chemical properties.

Soil pH was assayed with a pH electrode (InLab Science, Mettler Toledo, Switzerland) in soil water suspensions (1:2.5 weight/volume). The soil organic matter (SOM) content was assayed with acidified potassium dichromate (K_2_Cr_2_O_7_–H_2_SO_4_). The alkaline hydrolysis diffusion method was used to determine available soil nitrogen (AN). Available phosphorus (AP) was analyzed by the Olsen method (67). Available potassium (AK) was extracted with NH_4_OAc and analyzed by flame emission spectrometry. Available sulfur (AS) was analyzed with barium sulfate turbidimetry. Exchangeable calcium (ExCa) and magnesium (ExMg) were extracted with NH_4_OAc, exchangeable aluminum (ExAl) was extracted with KCl, and measured by the inductively coupled plasma (ICP) method (19).

### Soil DNA extraction and quantitative PCR (qPCR).

Total soil genomic DNA was extracted from 500 mg of fresh soil using a FastDNA spin kit (MP Biomedicals, United States) according to the standard protocol. The elution volume for DNA was 100 μL. The DNA was stored at −20°C for subsequent analyses.

We used quantified PCR (qPCR) to quantify the abundance of the pathogen R. solanacearum in the rhizosphere soil. R. solanacearum density was quantified by using specific primers FlicF (5′-GAACGCCAACGGTGCGAACT-3′)/FlicR (5′-GGCGGCCTTCAGGGAGGTC-3′) targeting the *f*liC gene coding the flagellum subunit (68). The qPCR analyses were carried out with a CFX96 Optical Real-time Detection System (Bio-Rad, United States). The reactions were conducted in a 20 μL mixture containing 10 μL of Pro Taq HS SYBR green (AG11701, Accurate Biotechnology, Hunan, Co., Ltd., China), 1 μL of each primer (10 μmol/L), 1 μL of template, and 7 μL of double-distilled water (ddH_2_O). The qPCR conditions were performed as described by Hu et al. (69) with some modification: 95°C for 5 min, followed by 30 cycles of 94°C for 30 s, 60°C for 40 s and 72°C for 30 s. Melting curve (60°C to 95°C, increment 0.5°C for 5 s) analysis was performed at the end of the PCR experiment to evaluate the specificity of the amplification. Standard curves were created using 10-fold serial dilutions (10^3^–10^7^) of a plasmid containing a copy of the *f*lic sequence. The coefficient of determination of the standard curve was 0.999, and the efficiency was 89.1%.

### Sequencing library construction.

16S rRNA high-throughput sequencing was performed for the rhizosphere soils in experiment I and experiment II. PCR amplifications were conducted with 515 forward primers (5′-GTGCCAGCMGCCGCGG-3′) and 806 reverse primers (5′-GGACTACHVGGGTWTCTAAT-3′), which amplified the V4 region of the 16S rRNA gene (70).

The PCR amplification conditions were as follows: 95°C for 3 min, followed by 27 cycles of 30 s at 95°C, 30 s at 55°C, and 45 s at 72°C, and a final extension was performed at 72°C for 10 min. PCR of 515F_806R was performed with 4 μL of 5 × *TransSt*art FastPfu buffer, 2 μL of 2.5 mM deoxynucleoside triphosphates (dNTPs), 0.8 μL of each primer (5 μM), 0.4 μL of *TransSt*art FastPfu DNA polymerase, 10 ng of extracted DNA, and ddH_2_O to a final volume of 20 μL. Agarose gel electrophoresis was performed to verify the size of the PCR amplicons. Amplicons were subjected to paired-end sequencing on the NovaSeq 6000 sequencing platform using PE250 chemical at Majorbio Bio-Pharm Technology Co. Ltd. (Shanghai, China).

A total of 2,274,014 (average read length was 256.15 bp) sequences were obtained from the 20 nondiseased soil samples in experiment I, and 898,729 (average read length was 256.17 bp) sequences were obtained from 8 diseased soil samples in experiment II. The raw reads were deposited into the NCBI short-reads archive database under accession number PRJNA804972 (experiment I) and PRJNA715361 (experiment II).

### Bioinformatics analysis.

After demultiplexing, the resulting sequences were merged with FLASH (v1.2.11) (71) and quality filtered with fastp (0.19.6) (72). Then, the high-quality sequences were denoised using the DADA2 (73) plugin in the QIIME2 (74) (version 2020.2) pipeline with recommended parameters, which obtains single nucleotide resolution based on error profiles within samples. DADA2 denoised sequences are usually called amplicon sequence variants (ASVs). Taxonomic assignment of ASVs was performed using the Naive Bayes consensus taxonomy classifier implemented in QIIME 2 and the SILVA 16S rRNA database (v138) for bacteria (threshold 0.7).

To complete the diversity and composition analyses, the sequence of each sample was rarefied to the lowest number of sequences (75). Rarefaction curves of ASVs were drawn to verify whether the sequencing depth was adequate to cover most microbial taxa. The differences of bacterial community structure among different soil pH were determined using analysis of similarities (ANOSIM) and principal-component analysis (PCoA) based on the weighted UniFrac or Bray-Curtis distance in the “vegan” package in the R. In order to identify the correlation between bacterial communities and environment variables, redundancy analysis (RDA) was determined in the vegan package.

Linear discriminant analysis (LDA) effect size (LEfSe) employed the factorial Kruskal–Wallis sum-rank test (α = 0.05) to identify taxa with significant differential abundances between categories (using all-against-all comparisons), followed by LDA to estimate the effect size of each differentially abundant feature (logarithmic LDA score ≥ 3.0). Significant taxa were used to generate taxonomic cladograms, which illustrated the differences between sample classes on the website http://huttenhower.sph.harvard.edu/galaxy.

### Microbial carbon source metabolic activity.

Microbial carbon source metabolic activity analysis was performed on the rhizosphere soil of pH 4.45, pH 5.90, pH 5.35, pH 5.60 and pH 6.45 in experiment I and the rhizosphere soil of CaO and control in experiment II. Microbial carbon source metabolism expressed in each Biolog EcoPlate (EcoPlate, Biolog, Hayward, CA, USA) was determined as average well color development (AWCD) (76). The experiment was performed on the day of rhizosphere soil sampling to avoid changes in microbial communities during storage of the soil. The carbon source utilization pattern for each soil sample was determined in accordance with the procedures described by Zhang et al. (77). The AWCD was calculated according to Wang et al. (78). The detailed carbon source usage was measured by the absorbance at 590 nm (79). And principal component analysis (PCA) was used to find the most related carbon sources within different treatments at 72 h of culturing (80). Carbon sources which had the arrow length above the average were selected to further present their well color development among different treatments.

### Statistical analyses.

The figures were created using GraphPad Prism 8.0.1. The mean and standard error for each set of data were calculated by independent-sample *t*-test (*P* < 0.05) or one-way analysis of variance (ANOVA) with Tukey’s honestly significant difference test (*P* < 0.05) were performed in SPSS software (version 17.0). Linear models to examine the relationships of pathogen abundance, disease incidence and potentially beneficial genera with soil pH in Origin 9. Spearman's rank correlation coefficient between the potentially beneficial genera with the R. solanacearum abundance was calculated using SPSS v17.0.
