# Supplementary material for: Alleviating Soil Acidification Could Increase Disease Suppression of Bacterial Wilt by Recruiting Potentially Beneficial Rhizobacteria
Source: Microbiol Spectr. 2022 Mar 7;10(2):e02333-21. doi: 10.1128/spectrum.02333-21 (PMC9045175; doi:10.1128/spectrum.02333-21)
Supplement: SUPPLEMENTAL FILE 1 — Supplemental material. Download SPECTRUM02333-21_Supp_1_seq12.pdf, PDF file, 0.7 MB [file spectrum02333-21_supp_1_seq12.pdf]

Supplementary Material

Alleviating soil acidification could increase disease suppression of bacterial wilt by recruiting potentially beneficial rhizobacteria

Shuting Zhang, Xiaojiao Liu, Lihua Zhou, Liyuan Deng, Wenzhuo Zhao, Ying Liu, Wei Ding*

College of Plant Protection, Southwest University, Chongqing 400715, China

*Corresponding author, Address: No.2 Tiansheng Road, Beibei, Chongqing, China.

Fax: +86 23 68250218. E-mail: dingw@swu.edu.cn

E-mail of all authors:

zhangshutinglj@163.com (S.Z)

xiaojiaoliu@email.swu.edu.cn (X.L)

1024497271@qq.com (L.Z)

153280434@qq.com (L.D)

578610705@qq.com (W.Z)

lyying1201@163.com (Y.L)

dingw@swu.edu.cn (W.D)

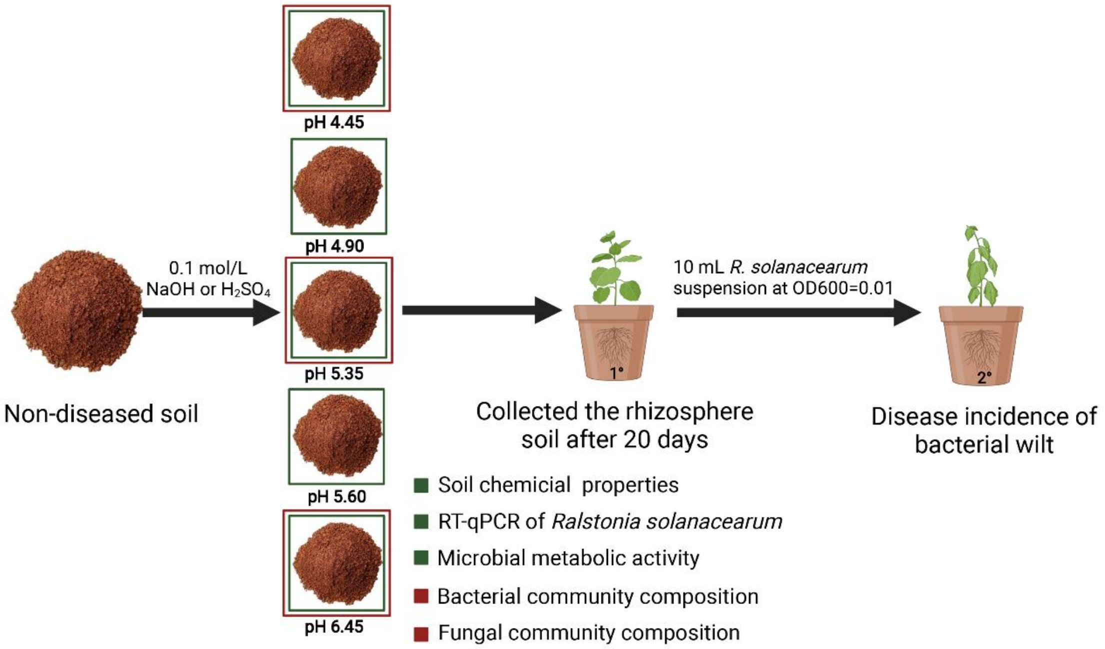

Fig. S1 Schematic representation of the acidification gradient experiment. Non-diseased soil from Pengshui was treated with 0.1 mol/L NaOH or H_2SO_4 to adjust the pH to different levels. Thirty days after treatment, the first population (1°) of *Nicotiana benthamiana* was planted (n=32 plants per treatment). After 20 days of growth, the rhizosphere soil of the plants was collected. Then, the second population (2°) of *Nicotiana benthamiana* was planted in bulk soil at different pH levels (n=24 plants per treatment). The disease incidence of bacterial wilt caused by *R. solanacearum* was quantified. The green and red boxes indicate the test items at different pH gradients.

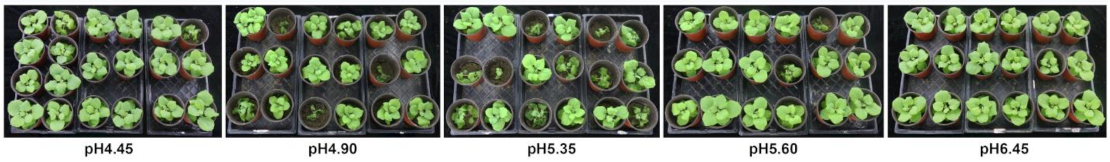

Fig. S2 The growth and occurrence of bacterial wilt in the second population (2°) of *Nicotiana benthamiana* at 10 days after inoculation with *Rolstonia solanacearum*

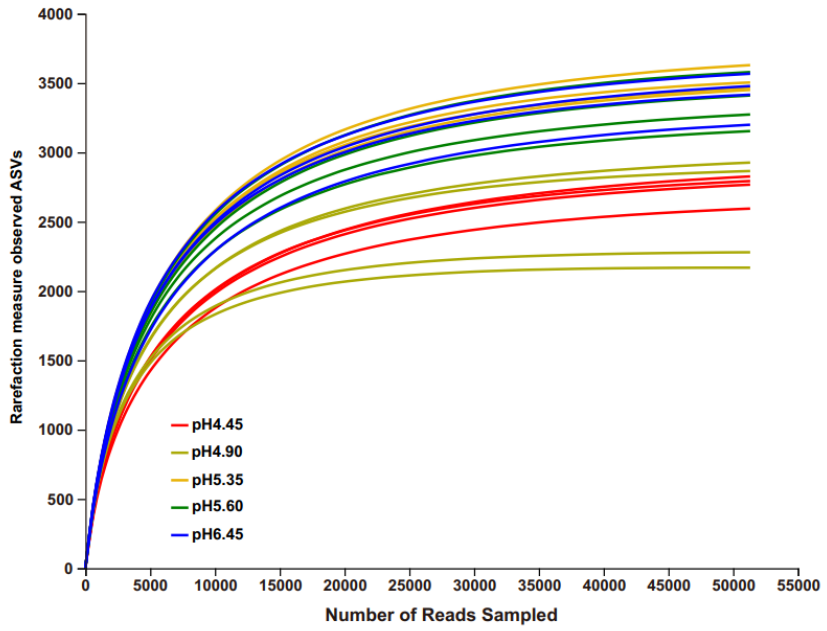

Fig. S3 The rarefaction curves of different treatments

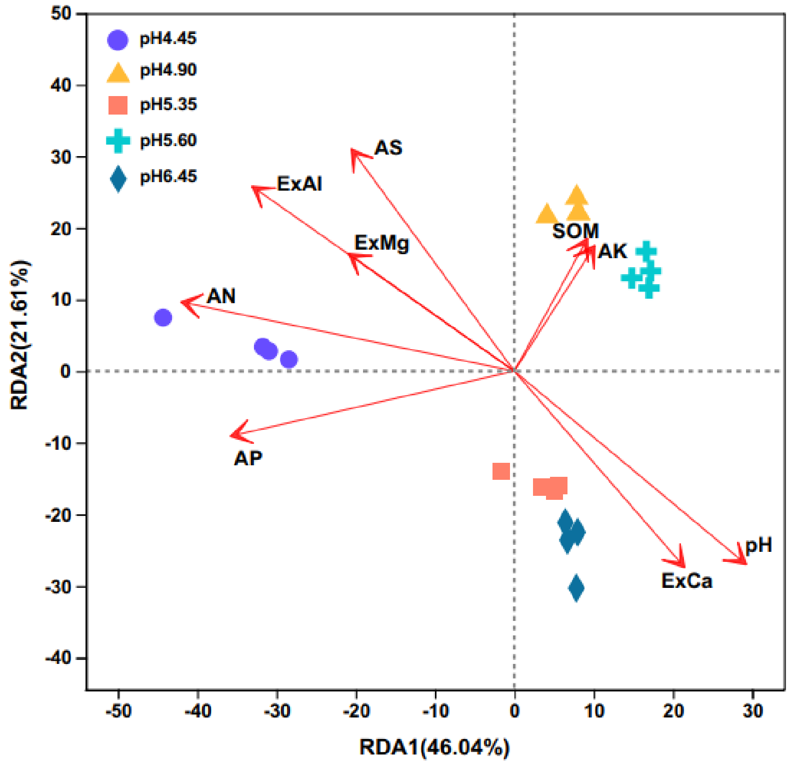

Fig. S4 Redundancy analysis (RDA) demonstrating the relationships between bulk soil environmental factors and rhizosphere soil bacterial (A) and fungal (B) community structures. AN, AP, AK and AS indicate available soil nitrogen, available phosphorus, available potassium and available sulphur, respectively. ExCa, ExMg and ExAl indicate soil exchangeable calcium, exchangeable magnesium and exchangeable aluminum, respectively

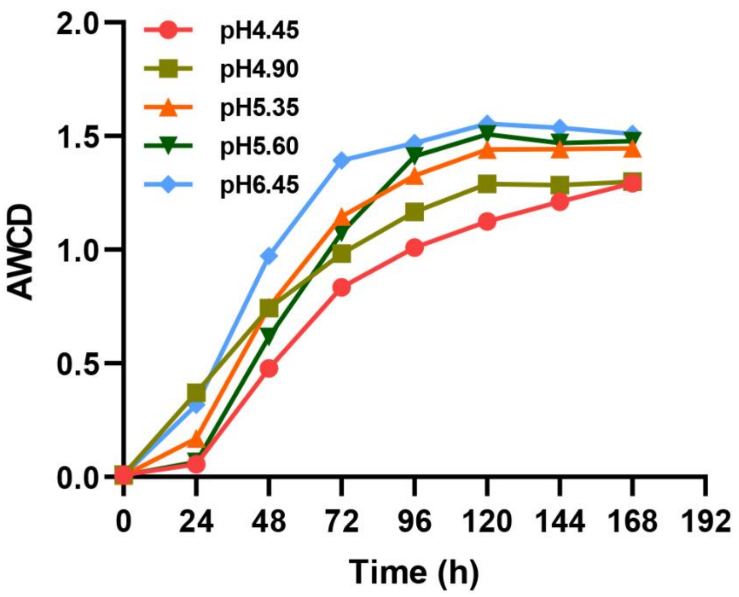

Fig. S5 Average well color development (AWCD) values of microbial communities at different soil Ph

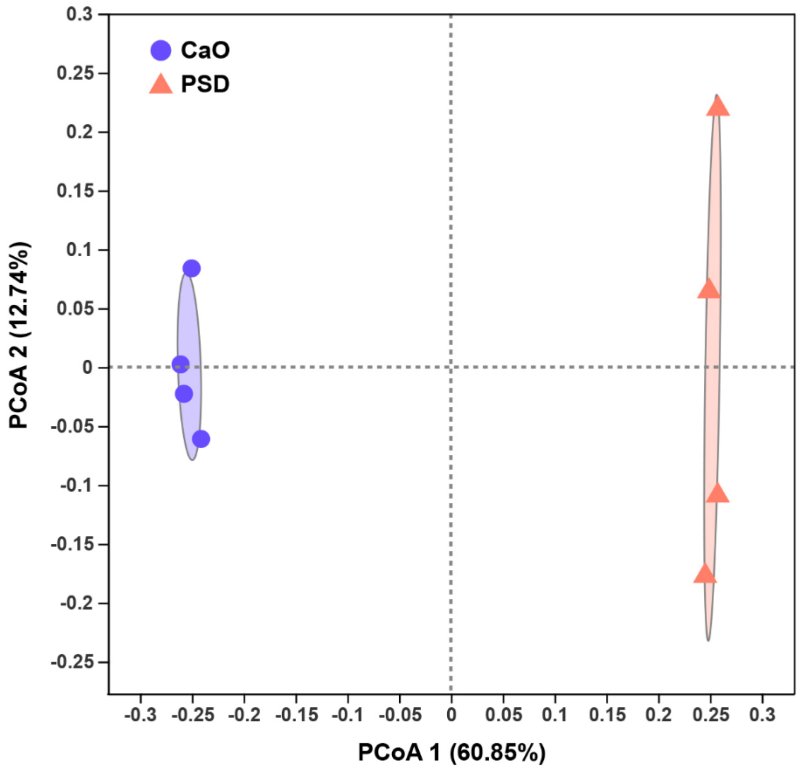

Fig. S6 The principal component analysis (PCoA, based on Bray-Curtis dissimilarity principal coordinate analysis) of bacterial community in CaO and control.

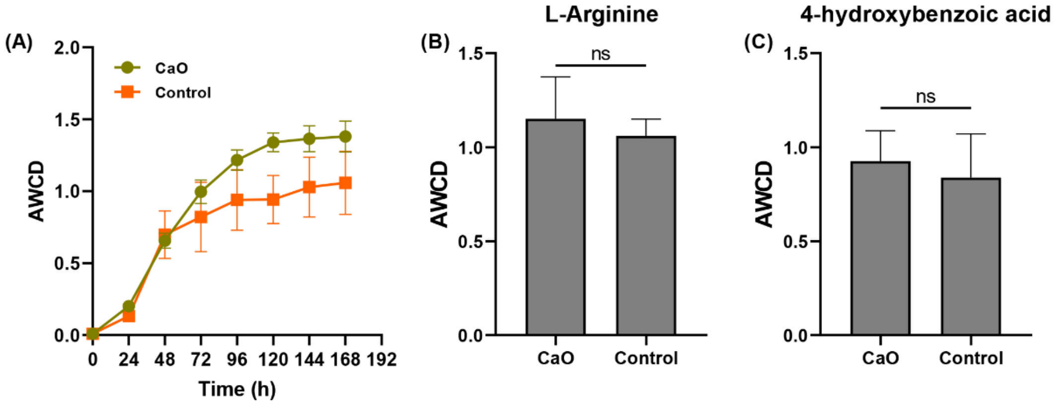

Fig. S7 Microbial carbon source metabolism capacity in CaO and control. A, Average well colour development (AWCD) values of microbial communities. B-C, The utilization of L-malic acid and 4-hydroxybenzoic acid at 72 h. The ns means no significant difference based on independent-sample t test.

Table S1 RDA parameters of different environmental factors and bacterial community composition

Environmental factors	R^2	*P*
AN	0.9295	0.001
ExAl	0.8811	0.001
pH	0.7899	0.001
AS	0.6885	0.001
AP	0.6708	0.002
ExCa	0.6012	0.001
ExMg	0.3451	0.026
SOM	0.2016	0.165
AK	0.1930	0.166

Note: AN, AP, AK and AS indicate available soil nitrogen, available phosphorus, available potassium and available sulphur, respectively. ExCa, ExMg and ExAl indicate soil exchangeable calcium, exchangeable magnesium and exchangeable aluminum, respectively
